# Derivational morphology reveals analogical generalization in large language models

**DOI:** 10.1073/pnas.2423232122

**Published:** 2025-05-09

**Authors:** Valentin Hofmann, Leonie Weissweiler, David R. Mortensen, Hinrich Schütze, Janet B. Pierrehumbert

**Affiliations:** ^a^Allen Institute for AI, Seattle, WA 98103; ^b^Paul G. Allen School of Computer Science and Engineering, University of Washington, Seattle, WA 98195; ^c^Center for Information and Language Processing, Ludwig Maximilian University of Munich, Munich 80538, Germany; ^d^School of Computer Science, Language Technologies Institute, Carnegie Mellon University, Pittsburgh, PA 15213; ^e^Oxford e-Research Centre, Department of Engineering Science, University of Oxford, Oxford, OX1 3QG, United Kingdom

**Keywords:** large language models, lexicon, analogy, linguistic rules, AI

## Abstract

What mechanisms underlie linguistic generalization in large language models (LLMs)? This question has attracted considerable attention, with most studies analyzing the extent to which the language skills of LLMs resemble rules. As of yet, it is not known whether linguistic generalization in LLMs could equally well be explained as the result of analogy. A key shortcoming of prior research is its focus on regular linguistic phenomena, for which rule-based and analogical approaches make the same predictions. Here, we instead examine derivational morphology, specifically English adjective nominalization, which displays notable variability. We introduce a method for investigating linguistic generalization in LLMs: Focusing on GPT-J, we fit cognitive models that instantiate rule-based and analogical learning to the LLM training data and compare their predictions on a set of nonce adjectives with those of the LLM, allowing us to draw direct conclusions regarding underlying mechanisms. As expected, rule-based and analogical models explain the predictions of GPT-J equally well for adjectives with regular nominalization patterns. However, for adjectives with variable nominalization patterns, the analogical model provides a much better match. Furthermore, GPT-J’s behavior is sensitive to the individual word frequencies, even for regular forms, a behavior that is consistent with an analogical account but not a rule-based one. These findings refute the hypothesis that GPT-J’s linguistic generalization on adjective nominalization involves rules, suggesting analogy as the underlying mechanism. Overall, our study suggests that analogical processes play a bigger role in the linguistic generalization of LLMs than previously thought.

## Significance

Large language models (LLMs) are a type of artificial intelligence technology that is currently being deployed in a rapidly growing range of applications. The sensitive nature of some of these applications makes it imperative that we have a precise understanding of the inner workings of LLMs. By uncovering the role of analogical mechanisms for the linguistic generalization of LLMs, our study contributes to this goal and casts a light on their impressive language skills. Furthermore, the results of our experiments have the potential to indicate pathways for further improving LLMs.

In the recent past, large language models (LLMs) such as Chinchilla (1), Gemini (2), GPT-4 (3), LLaMA (4), Mistral (5), OLMo (6), and PaLM (7) have reached an unprecedented level of linguistic capability. While some have likened the language skills of LLMs to those of humans (8, 9), others have highlighted the persistent linguistic inadequacies of LLMs (10–13). Crucially, however, it is well established that they go beyond simply copying from the training data (14–19). With the ability to generate and process novel expressions being widely viewed as a hallmark of human intelligence, the controversy around the extent to which the language skills of LLMs are human-like has sparked a wider discussion about whether AI is finally truly intelligent.

What are the mechanisms underlying linguistic generalization in LLMs? Are they human-like? Prior studies have approached this question by investigating the extent to which the language skills of LLMs resemble abstract, symbolic linguistic rules (20, 21). For instance, the consistency with which LLMs provide the correct agreement marking for unseen subject–verb pairs has been interpreted as evidence that they implicitly infer a set of rules from the training data (18). Rules are a form of generalization that results from language learners scanning the available data and distilling abstract knowledge about the linguistic patterns exhibited in the data. Each rule has a structural description, which specifies what properties must be met for the rule to apply, and a structural change, which specifies how the input is changed to produce the output. When processing novel, previously unseen input, the rule matching the input properties is selected, and applied to produce the output.

Much less attention has been devoted to the question of whether the language skills of LLMs could be the result of analogical processes operating on stored exemplars. Typically expressed in the form A:B::C:D (“*A* is to *B* as *C* is to *D*”), an analogy is an assertion that the relation of *A* to *B* is similar to the relation of *C* to *D*. For example, the analogy *moon*:*planet*::*planet*:*sun* makes a generalization about our solar system, and entertaining this analogy was an important step in the Copernican development of the heliocentric theory. Analogical models of linguistic generalization take the *D* position in the template to be an unknown and add a level of statistical inference to find the optimal way to fill the *D* position. Specifically, for a probe *C* with unknown *D*, they ask which of the potential completed analogies for *C* has the best statistical support, as defined by the behavior of exemplars in memory that are similar to *C* (referred to as the neighborhood of *C*). Analogical models of word formation have proved successful in capturing detailed patterns of variation in multiple languages (22–28). For example, an analogical model can explain why the past tense of an English nonce verb *spling* is often judged to be *splang* (even though an *-ed* past tense is more common), based on the behavior of verbs in its neighborhood such as *sing*, *ring*, and *sink* (27).

Within cognitive science, analogical generalization is argued to be a central learning mechanism and a foundation for the ability of humans to form abstract conjectures (29–32). In evaluating the LLMs’ analogical capabilities, we thus share with prior work the goal of evaluating the LLMs’ capabilities for implicit abstraction. However, there are significant differences between analogical and rule-based theories of human reasoning, particularly with regard to effects of frequency. By generalizing on the fly over stored exemplars, analogical models have both the forest and the trees, in the form of both generalizations (forests) and trees (individual exemplars). As a result, frequency effects at multiple levels from individual known words to the overall prevalence of different patterns are predicted. Frequency effects do appear in rule-based theories in that the learner must encounter a sufficient mass of examples to learn a rule in the first place. However, the mental lexicon is only a repository of unpredictable information, which means that once a regular rule has been learned, its outputs are not stored. For example, the lexicon would include *rotate*, since the association of this word form with this specific concept could not have been predicted. However, it does not include the regular past tense *rotated*. The frequency of *rotated* would not be available in the model, neither as a factor in forming the past form of *rotate*, nor as an influence on the output for other base forms. This central difference between analogical models and rule-based models means that frequency effects are a repeated theme in the sections below.

LLMs store a considerable amount of their training data in their model weights (19, 33–35), thus implicitly providing a reservoir of stored exemplars that might support analogical reasoning as a mechanism for all generalizations. However, it is also possible that the models memorize examples but generalize only via rules. A third possibility is that LLMs learn rules for regular linguistic phenomena, while handling irregular linguistic phenomena by means of analogy over stored exemplars, in line with dual-mechanism approaches (27, 36–38). Thus, the way that the stored data are used in generalizing by LLMs is an open question.

Here, we present in-depth analysis of the role of analogical linguistic generalization in LLMs. Our work is motivated by a key shortcoming of the existing literature: Prior studies, most of which explore rule-based generalization in LLMs (e.g., ref. 18), have focused on syntactic phenomena such as subject–verb agreement, which display a high degree of regularity. Crucially, in such cases, both rule-based and analogical, exemplar-based approaches make the exactly same predictions (39–41); in other words, rule-like behavior of LLMs on regular linguistic phenomena does not represent any evidence for rule-based generalization. This very insight was at the heart of the pioneering research that first applied neural networks in the context of language learning, which argued that “lawful behavior and judgments may be produced by a mechanism in which there is no explicit representation of the rule” (42). In fact, neural network models of language depend in important ways on similarity relations among input examples (43–47), suggesting that analogy might play a major role for the language skills of LLMs. This hypothesis has not been systematically tested previously.

We focus on a domain of language that is known to exhibit more variability than syntax, making it better suited for distinguishing rule-based from analogical generalization: derivational morphology (48–52). Specifically, we analyze how LLMs learn English adjective nominalization with *-ity* and *-ness* (53–56), focusing on adjectives that themselves contain a derivational suffix (e.g., *available*, *selfish*, *hyperactive*). Such cases of affix stacking are an ideal testbed for our purposes since the adjective class (i.e., the adjective-final suffix) provides a controlled way to vary the regularity of the nominalization process: While some adjective classes are nominalized in a very regular way, exhibiting a clear preference for either *-ity* (e.g., adjectives ending in *-able* such as *available*) or *-ness* (e.g., adjectives ending in *-ish* such as *selfish*), others exhibit a substantial degree of variability (e.g., adjectives ending in *-ive* such as *hyperactive*). Furthermore, English adjective nominalization with *-ity* and *-ness* has been shown to be fully explainable as a result of analogical generalization in humans (57), suggesting that LLMs might employ the same mechanism. In general, probabilistic models (58), and particularly exemplar-based analogy models (59, 60), have recently proven very successful at modeling competition between nearly synonymous linguistic structures, which is an additional motivation for our work. While there has been some previous work on the morphological capabilities of LLMs (e.g., refs. 13, 15, and 61–63), it has not diagnosed the generalization mechanisms underlying those capabilities.

As a key contribution of our work, we introduce a method for probing the generalization mechanisms underlying the language skills of LLMs: We fit cognitive models that instantiate certain generalization mechanisms to the LLM training data and compare their predictions on unseen data with those of the LLM. This approach, which is inspired by a long line of research in computational psychology using computer simulations (64–66), adds to the growing body of work that seeks to explain the behavior of LLMs as a result of the data on which they were trained (18, 67–70). Our method also informs the LLMs that we analyze. Our primary target is GPT-J (71). GPT-J is one of the GPT series of generative pretrained transformer models, and it is one of the few LLMs whose training data, namely the Pile (72), is publicly available. We also present some results on GPT-4, as an example of a state-of-the-art model, even though lack of access to its training data creates some limitations. For the sake of convenience, we provide short definitions of the technical terms used throughout the paper in Table 1.

**Table 1.** Key technical terms used in the paper, as they apply to the domain of word-formation

Adjective class	Set of adjectives ending in the same suffix.
Adjective nominalization	Derivational morphology that converts adjectives to nouns.
Analogy	Inference of a new word form *D* from word forms *A*, *B*, and *C* such that *C* is phonologically similar to *A*, and *D* is to *C* as *B* is to *A*.
Derivational morphology	Operations that change the meaning or part of speech of a word.
Derivative	Word form obtained by applying derivational morphology.
Exemplar	A specific instance of an item that is stored in memory.
Frequency	Count of a word or set of words in the corpus.
Neighborhood	Exemplars with a high phonological similarity to a probe.
Nonce word	Pseudoword invented for the purposes of an experiment.
Probability	Likelihood of a form as computed by a model.
Probe	Word form for which a derived word form is to be generated.
Rule	Statement of a pattern in which the output depends only on a symbolic description of the input.

## Results

### Generalization to Nonce Words.

We compare the linguistic generalization behavior of GPT-J with that of two high-performing cognitive models: the Minimal Generalization Learner (MGL; 73, 74) and the Generalized Context Model (GCM; 29, 30, 75). The MGL is a rule-based model that we have selected because it undertakes to capture detailed patterns of variation that earlier rule-based models did not capture, by assigning statistical reliability to rules. The GCM is an exemplar-based analogy model that was developed for perceptual categorization, and then successfully adapted to variability in inflectional morphology (24, 27). The two models are similar in that both generalize over word pairs consisting of a base form and a derived form, and generate predictions for a novel derived form by mapping the phonological form of the base to the phonological form of the derivative. They can be trained on either word types or word tokens. The inventory of word types corresponds to the list of words in a mental lexicon; only the existence of a word in the language is taken into account, and not its frequency in the training data. In an inventory of word tokens, each occurrence of a word in the training data is treated separately, with the result that more frequent words have more instances than less frequent words. We consider both settings since the contrast between behaviors governed by type frequencies and those governed by token frequencies is a major theme in cognitive research on the lexicon (e.g., ref. 74).

The mechanics of GPT-J is substantially different. GPT-J has been trained on a large corpus of text, encoded as sequences of words and subunits of words (e.g., individual characters). The input and output of GPT-J both consist of text that can span several hundred words. To probe the implicit world knowledge of a model such as GPT-J, we can ask it to generate text answering questions about real-world facts. Similarly, to probe the model’s implicit knowledge of derivational morphology, we can ask it to answer questions about the derived forms corresponding to a variety of base forms.

We focus on English adjective nominalization and examine four adjective classes (i.e., sets of adjectives ending in the same suffix), two of which clearly prefer *-ity* or *-ness* (specifically, adjectives ending in *-able* or *-ish*), and two of which are less regular while still showing an overall tendency toward one of the two suffixes (specifically, adjectives ending in *-ive* or *-ous*). We train the cognitive models on all adjective–derivative pairs that meet the following three criteria: i) the adjective belongs to one of the four adjective classes in question; ii) the derivative ends in *-ity* or *-ness*; iii) both the adjective and the derivative occur in the Pile.

For evaluation, we use UniPseudo (76) to generate 50 nonce adjectives for each of the four adjective classes. We check that both the generated nonce adjective and the two corresponding derivatives have a frequency of zero in the Pile, i.e., they have never been seen by either the cognitive models or GPT-J, thus providing an ideal test set for probing linguistic generalization. We then feed all nonce adjectives into the cognitive models and determine which of the two competing derivatives they prefer. For GPT-J, we measure the probability that it assigns to the two derivatives resulting from adding *-ity* and *-ness* to the adjectives. Specifically, we use GPT-J’s autoregressive language modeling head to compute the log probabilities for the subword units into which the derivatives are split and sum them. We take the derivative with the higher total log probability as the preferred one. Since prior research has shown that varying prompts (i.e., the texts used to elicit LLM responses) can heavily affect LLM behavior (77), we repeat this procedure with 12 different prompts (*SI Appendix*, Supporting Text). If not stated otherwise, the presented results are averaged over prompts.

**Fig. 1.**
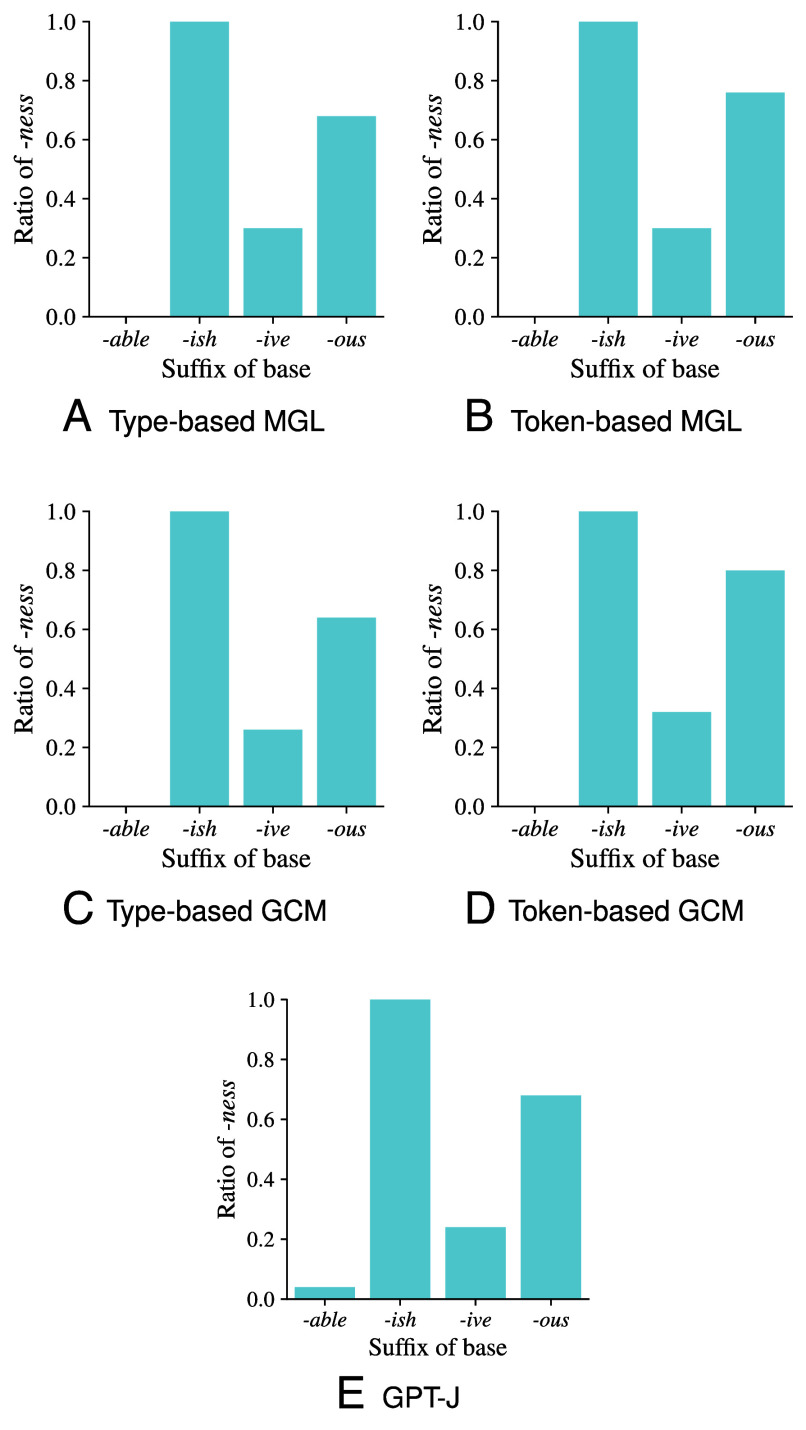
Distribution of preferred nominalization type (specifically, ratio of *-ness* derivatives) for unseen nonce adjectives, for rule-based models (*A* and *B*), exemplar-based models (*C* and *D*), and GPT-J (*E*). Models based on types are shown on the *Left* (*A* and *C*), and models based on tokens are shown on the *Right* (*B* and *D*). The ratio is computed as the number of *-ness* predictions divided by the total number of predictions (i.e., *-ness* and *-ity* predictions).

As shown in Fig. 1, both MGL and GCM—in the type-based as well as the token-based setting—make completely consistent predictions for the two adjective classes that strongly prefer one affix. They always predict *-ity* for *-able* and *-ness* for *-ish*. Thus, both cognitive models reproduce the regular behavior that characterizes these two adjective classes. GPT-J also predicts *-ity* for *-able*, and it predicts *-ness* for *-ish* in all but two cases for just one of the prompts (*turgeishity* and *prienishity*). GPT-J is nearly as successful in capturing the regular cases as MGL and GCM, and these in turn match the predictions of GPT-J equally well (Table 2, *Upper* panel). Thus, the regular adjective classes do not tell us whether GPT-J is more like a rule-base model or an analogical model.

**Table 2.** Comparison with cognitive models

		Examples		Counts		MGL		GCM	
Regularity	Suffix	Real	Nonce	*-ity*	*-ness*	Type	Token	Type	Token
High	*-able*	available	tegornable	11,081	1,034	0.893	0.893	0.893	0.893
	*-ish*	selfish	friquish	0	1,502	0.997	0.997	0.997	0.997
Low	*-ive*	sensitive	cormasive	4,508	2,438	^†^0.658	0.662	^†^0.622	**0.688**
	*-ous*	luminous	momogorous	1,372	2,450	^†^0.657	^†^0.613	^†^0.610	**0.703**

The table shows real and nonce examples for the four examined adjective classes, the counts of corresponding derivatives in the Pile as well as the results of rule-based and exemplar-based analogy models evaluated against GPT-J. Specifically, the choice of *-ity* or *-ness* for each nonce word by each of the four models shown is compared to GPT-J’s choice for that item. The evaluation measure is accuracy, i.e., the percentage of the model’s choices that matched GPT-J’s choice. We highlight the highest accuracy value (i.e., the best-matching cognitive model) in each row in boldface—for the two adjective classes where there is a winner (i.e., *-ive* and *-ous*), this is the token-based GCM model. We highlight accuracy values that are significantly (*P* < 0.05) worse than the highest accuracy value in each row with a ^†^.

Moving to the two adjective classes that show more variability between *-ity* and *-ness* (i.e., *-ive* and *-ous*), both MGL and GCM generate variable outcomes with a higher rate of *-ness* for *-ous* than for *-ity* (Fig. 1). However, the predictions differ substantially in detail: the cognitive models (in the type-based as well as the token-based setting) agree in only 54% of the adjective types. Crucially, the cognitive model that matches the predictions of GPT-J on these two adjectives classes best is the token-based GCM model (Table 2, *Lower* panel). As a concrete example, we consider the nonce adjective *pepulative*. The MGL models map *pepulative* to a rule that prescribes *-ity* following *-tive*, which in the type-based as well as the token-based setting has the highest confidence of all competing rules and is hence selected by both MGL models. The GCM models, by contrast, are more strongly influenced by local similarity effects. While overall there are a larger number of *-ity* derivatives in the neighborhood of *pepulative* (e.g., for adjectives ending in *-lative* there are 88 derivatives with *-ity* vs. 27 with *-ness*), many of the adjectives particularly close to *pepulative* have *-ness* derivatives with a high token frequency (e.g., *manipulativeness* has a token frequency of 1,544 vs. 26 for *manipulativity*). This difference is reflected by the GCM models, where the type-based model predicts *-ity*, but the token-based model predicts *-ness*. GPT-J, on the other hand, prefers *-ness* for this example and hence matches the behavior of the token-based GCM model.

Our results show that the generalization behavior of LLMs on linguistic phenomena with a high degree of variability is best explained as a result of analogical mechanisms. This finding is in line with the observation that LLMs store a considerable amount of their training data in their model weights (19, 33–35), and it further suggests that these stored data actively contribute to the language skills displayed by LLMs. Our results are consistent with a model that generalizes all adjective nominalizations by analogy; they eliminate the possibility that all nominalizations are generated by rules. However, there remains the possibility that LLMs effectively use analogies in cases of variation and apply rules for adjective classes with a high degree of regularity. This possibility is suggested by earlier theories of inflectional morphology, proposing dual-mechanism models in which regular plurals and past tenses are created by rules, while irregular forms involve analogies (36–38). To address this possibility, it is necessary to look into frequency effects for individual words, as discussed in prior work (78, 79). We will do so in the next sections.

### Predictions for Seen Words.

According to cognitive theories, analogies are based on remembered examples. If the mechanism underlying GPT-J’s behavior is analogical, it must implicitly remember a large number of examples. As the first step in evaluating this inference, we ask how well GPT-J’s behavior matches the frequencies of words seen in its training data. Accurately matching the training data, derivative by derivative, would imply that the distributed representations in GPT-J encode information about individual derivatives.

We extend the four adjective classes examined so far and include six other adjective classes that can be nominalized with either *-ity* or *-ness*: *-al*, *-ar*, *-ed*, *-ic*, *-ing*, and *-less*. We can divide the ten adjective classes into four groups with similar degrees of competition between *-ity* and *-ness* (*SI Appendix*, Table S2):

*-ed*, *-ing*, *-ish*, *-less* (R-NESS): This group exhibits the highest degree of regularity and almost always takes *-ness*.
*-able*, *-al*, *-ar*, *-ic* (R-ITY): This group also exhibits a high degree of regularity (although somewhat lower than in the case of R-NESS), with a strong tendency toward *-ity*.
*-ous* (V-NESS): This adjective class exhibits a high degree of variability, with a slight tendency toward *-ness*.
*-ive* (V-ITY): This adjective class also exhibits a high degree of variability, with a slight tendency toward *-ity*.

We ask whether GPT-J treats adjectives from these four groups differently, and whether differences between the more regular and more variable ones correspond to differences in the training data. We draw upon the Pile and extract all derivatives ending in *-ity* and *-ness* whose bases belong to one of the 10 adjective classes. To decrease noise, we only extract derivatives whose bases also occur in the Pile and apply several filtering heuristics, such as excluding words with nonalphabetic characters. To include all productively formed derivatives, we do not impose a frequency threshold on the derivatives.

The overall setup of probing GPT-J is identical to the comparison with the cognitive models: We measure the probability that GPT-J assigns to the two derivatives resulting from adding *-ity* and *-ness* to the adjectives, using the same set of prompts. Following this procedure, we evaluate GPT-J on all 48,995 bases from the Pile. If not stated otherwise, results are again averaged across prompts.

**Fig. 2.**
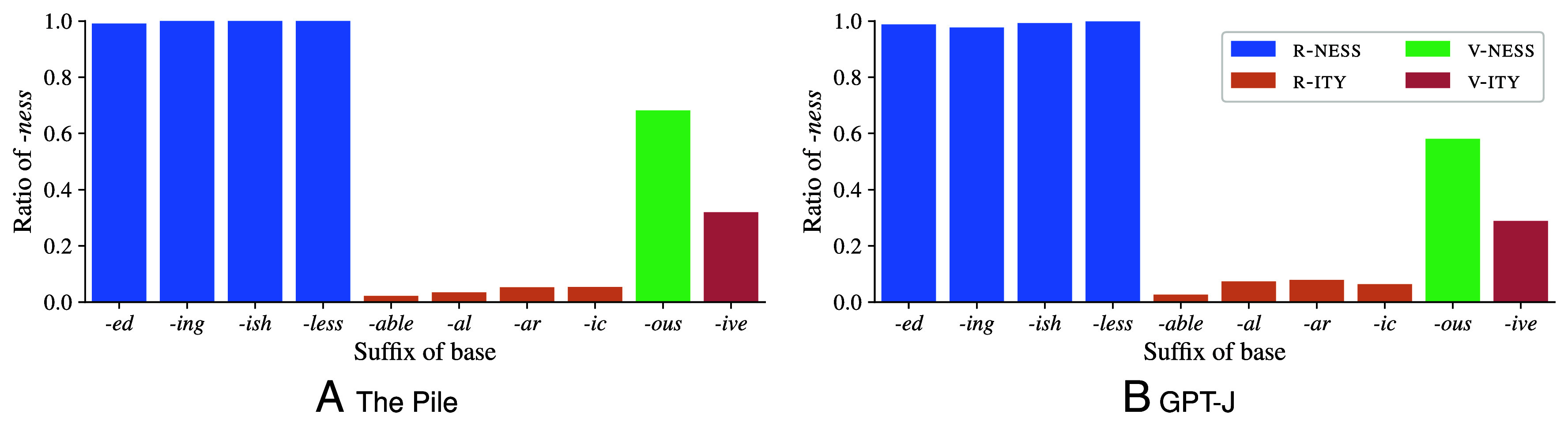
Ratio of bases preferring *-ness* in the Pile (*A*) and GPT-J’s predictions with one example prompt (*B*). Results are similar for the other prompts. The suffixes of the base (i.e., adjective classes) are grouped by degree of competition between *-ity* and *-ness*.

Fig. 2 compares, for each adjective class, the ratio of bases for which GPT-J prefers *-ness* compared to *-ity* with the statistics from the Pile. We find that the two distributions are very similar: almost no competition for the bases in R-NESS (i.e., *-ed*, *-ing*, *-ish*, *-less*), little competition for the bases in R-ITY (i.e., *-able*, *-al*, *-ar*, *-ic*), and strong competition for V-NESS (i.e., *-ous*) and V-ITY (i.e., *-ive*). The tendency toward *-ity* and *-ness* is also exactly as predicted based on the training data—the average correlation between the class-level *-ity*/*-ness* ratios in the training data (Fig. 2*A*) and GPT-J predictions (Fig. 2*B*) is 0.995 (±0.004; *P* < 0.001 for all prompts), measured using Pearson’s *r*. For multiple comparisons, *P*-values are corrected using the Holm–Bonferroni method (80).

Furthermore, GPT-J matches the training data statistics even on the level of individual bases: Across all bases, the accuracy of GPT-J’s preference for one of the two derivatives compared against the training data (considered here as the ground truth) is 89.5% (±4.8%); the derivative preferred by GPT-J is generally the derivative that is more likely in the training data.

Table 3 shows that there is variation between individual adjective classes, with bases in R-NESS (*-ed*, *-ing*, *-ish*, *-less*) having above 95% accuracy, bases in R-ITY (*-able*, *-al*, *-ar*, *-ic*) having above 85% accuracy, and bases in V-ITY (*-ive*) and V-NESS (*-ous*) having below 85% accuracy, but the general level of agreement is very high.

**Table 3.** Match between preferred derivatives in the training data and derivatives preferred by GPT-J

Adjective class	Suffix	Accuracy
R-NESS	*-ed*	0.986 ± 0.007
	*-ing*	0.989 ± 0.014
	*-ish*	0.995 ± 0.004
	*-less*	0.999 ± 0.001
R-ITY	*-able*	0.896 ± 0.082
	*-al*	0.884 ± 0.073
	*-ar*	0.896 ± 0.060
	*-ic*	0.867 ± 0.090
V-NESS	*-ous*	0.788 ± 0.038
V-ITY	*-ive*	0.842 ± 0.012

Thus, GPT-J’s morphological preferences closely mirror the statistics of the data it was trained on. The fact that GPT-J very consistently prefers the derivative with the higher frequency in the training data, even in cases such as adjectives ending in *-ive* where the suffix alone is a bad predictor of *-ity* vs. *-ness*, suggests that it stores many derivatives in its model weights. This is again in line with an analogical mechanism.

However, it is still possible that some of the high-regularity adjective classes (e.g., *-ish*) are handled by a rule, as suggested by dual-mechanism approaches. Next, we will disentangle these two hypotheses.

### Frequency Effects and Neighborhood Effects.

To further test whether at least part of GPT-J’s behavior on adjective nominalization can be explained by rules, we analyze the extent to which GPT-J prefers an observed nominalized form over an alternative, nonobserved nominalized form. We consider only cases in which just one outcome of nominalization is attested in the Pile and measure the difference in the log probability that GPT-J assigns to the attested vs. the unattested form. This difference can be viewed as reflecting GPT-J’s confidence in using a form that it has encountered during training; a large difference indicates high confidence, while a small difference reflects low confidence. For each adjective class, we create two sets: one in which the attested derivative has a low frequency in the Pile, f∈(0,10], and one in which the attested derivative has a high frequency in the Pile, f∈(100,∞).

If an adjective class is handled by a rule, the difference in frequency between the two sets should not affect GPT-J’s confidence in predicting the attested derivative. This is because rule-based theories abstract away from individual words; once a rule has been acquired, regular complex forms are assumed to be generated on the fly, much like complex sentence structures are, rather than being stored in memory. Does this match GPT-J’ behavior for any of the adjective classes? We operationalize this question by i) measuring GPT-J’s confidence (i.e., the log probability difference between the attested and the unattested derivative) for low-frequency derivatives with f∈(0,10], and ii) measuring the relative increase in confidence for high-frequency derivatives with f∈(100,∞). If an adjective class is handled by a rule, this relative increase should be zero. We again divide the adjective classes into the four regularity-based groups defined above (R-NESS, R-ITY, V-NESS, and V-ITY).

**Fig. 3.**
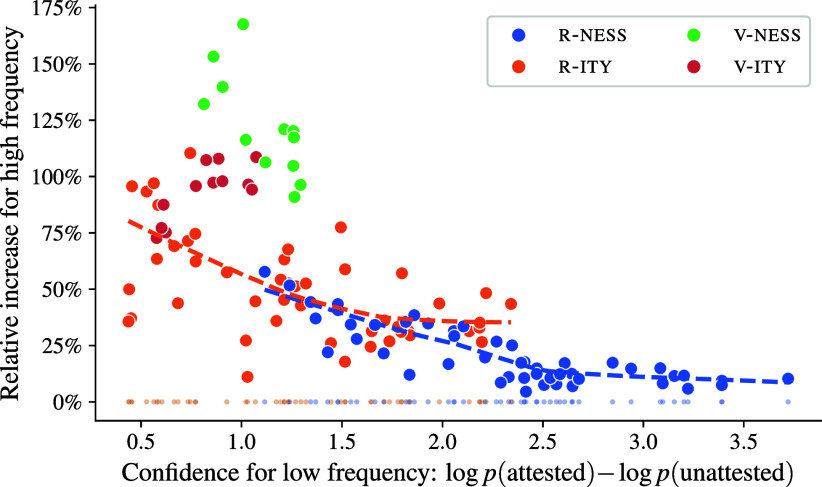
Impact of word frequency on GPT-J’s confidence in its choice. x-axis: Log probability difference between the attested and unattested choices for low-frequency derivatives with f∈(0,10]. We have converted the log probabilities from base *e* to base 10 for better readability. y-axis: Relative increase in confidence for high-frequency derivatives with f∈(100,∞). Each dot corresponds to GPT-J’s predictions for an adjective class given a specific prompt. Dots are colored by degree of competition between *-ity* and *-ness*. We added LOWESS lines for R-NESS and R-ITY. Dots at *y* = 0% indicate the expected behavior if R-NESS and R-ITY were handled by rule.

Fig. 3 displays the results. The relative increase in confidence is positive for all adjective classes and for all prompts, indicating that GPT-J is always more confident in its decision for the frequent than the rare derivatives, even for the R-NESS class. This indicates that the model has stored distributed representations for all the derivatives, contrary to the predictions of dual-mechanism models. Put differently, none of the adjective classes are handled by rule.

Overall, the examined adjective classes exhibit a downward slope in Fig. 3, which is also reflected by the R-NESS and R-ITY groups individually (indicated by trendlines). Thus, the more confident the model was in its decisions for the low-frequency group, the smaller the effect of word frequency on confidence. This finding is difficult to explain in a rule-based model, but it is perfectly in line with analogy as the underlying generalization mechanism.

More specifically, we will attribute the downward slope to the fact that the neighborhoods for probes from different adjective classes show varying degrees of competition between *-ity* and *-ness*. For the most regular adjective classes on the right-hand side of Fig. 3, there is little competition between *-ity* and *-ness* in the neighborhood for any given probe; for example, for an adjective ending in -ish, all adjectives in its neighborhood are nominalized with *-ness* (cf. Table 2), and hence the model confidence is high even if the attested derivative has a low frequency. In other words, if a derivative is strongly encoded in the model weights due to high frequency in the training data, this does not increase model confidence, because a highly homogeneous neighborhood already provides a clear signal as to which form should be preferred.

Lower confidence levels for the low-frequency forms toward the left of Fig. 3 represent cases in which the neighborhood of the probe is more heterogeneous. Here, the neighborhood alone provides a less clear signal as to which of the two alternative derivatives should be preferred; for example, for an adjective ending in *-ive*, its neighborhood often contains both adjectives nominalized with *-ness* and adjectives nominalized with *-ity* (cf. Table 2). As a result, the frequency in the training data becomes a critical factor for model confidence. If the frequency of the attested derivative is low, it is only weakly encoded in the model weights. Consequently, the model must rely on a heterogeneous neighborhood, which results in low confidence. On the other hand, if the frequency of the attested derivative is high, the resulting encoding in the model weights is strong, thus providing a clear signal beyond the neighborhood and leading to high confidence.

To quantify the neighborhood effect, we calculate the Shannon entropy of the distribution over *-ity* and *-ness* as the preferred form in the Pile for each adjective class. This serves as a rough approximation of the competition between *-ity* and *-ness* that is expected to exist in the neighborhoods of probes from each adjective class. We then use Pearson’s *r* to measure the correlation between the entropy and the confidence increase for high-frequency derivatives. At $r^{2}=0.75$, *P* < 0.001, this correlation is highly significant. Thus, the more heterogeneous the neighborhoods of probes from an adjective class, the greater the impact of the attested derivative’s frequency on GPT-J’s confidence—a finding that is exactly in line with the predictions of analogical models (e.g., ref. 81) while being completely at odds with rule-based approaches, which do not assume such frequency effects to begin with.

The left side of Fig. 3 exhibits more variability than the right side. We believe that this variability is caused by local neighborhood effects and the interaction of these effects with the prompting mechanism. Recall from the discussion of the nonce word *pepulative* that analogical models are sensitive not merely to the overall statistics for the two competing nominalizations, but also to the similarity and frequency of the most similar neighbors. These localized effects—which for the case of attested derivatives would also include semantic similarity—create a lumpy prediction landscape whose properties we do not try to quantify here. Meanwhile, the prompting mechanism is known to influence the focus and bias of the underlying transformer model (82). Slightly different prompts direct the focus toward different parts of the lumpy landscape, and would hence produce noise in the datapoints for Fig. 3.

To sum up, our analysis suggests that GPT-J learns adjective nominalization by implicitly storing derivatives in its model weights. In cases where the exemplar neighborhood for a probe is highly homogeneous, GPT-J produces highly regular outputs. While regular, or rule-like, behavior of LLMs has been observed before (e.g., ref. 18), our results contextualize this finding in important ways, suggesting that rule-like behavior forms the end of a gradient characterized by varying levels of regularity. This result is not consistent with assuming a qualitative difference between forms derived by rule and stored exemplars. However, it is exactly in line with the predictions of exemplar-based analogy models (e.g., refs. 24, 27, and 28).

### Human Use of Word Types vs. Tokens.

We have established that GPT-J relies on token-level analogical generalization. In contrast, previous studies have concluded that humans generalize over word types (83–85): Their propensity to generalize a word formation pattern depends on the number of distinct word types in the individual’s mental lexicon that support the pattern (referred to as the size of the lexical gang). This points to a difference between the morphological processing in humans and LLMs. We will now investigate this difference in greater detail, by comparing the predictions of GPT-J to judgments made by humans.

#### Judgments of nonce words.

First, we make a direct comparison to GPT-J’s behavior for nonce words. 22 native English speaker volunteer annotators indicated their preference for the *-ity* vs. the *-ness* derivative of each nonce adjective in our study. Because GPT-J is not a state-of-the-art model, we also introduce an additional comparison, by asking whether a more recent model is more human-like in its judgments. Specifically, we evaluate GPT-4 (3) on the same set of adjectives. If GPT-4 displays more human-like judgments than GPT-J, then the trend of improving LLMs through larger training sets and bigger model sizes will have paid off in this domain.

In Table 4, we take the derivative more often selected by humans as the ground truth. The table gives the accuracy of GPT-J, GPT-4, as well as the cognitive models considered above (i.e., MGL and GCM), measured against this human response. The type-based GCM model overall matches the human behavior best. While all cognitive models perfectly reproduce the homogeneous behavior for *-able* and *-ish*, the type-based GCM model better matches the human predictions for *-ive* and *-ous*, as reflected by large gaps compared to the second-best model, type-level MGL (*-ive*: 4%, *-ous*: 8%). The token-based variants of MGL and GCM match the human behavior substantially worse than the type-based variants, which is exactly in line with what has been suggested in prior work (74, 84). Moving to the results for GPT-J, it turns out to match the human responses worse than any of the cognitive models, for all four adjective classes. The gap compared to the best cognitive model, type-based GCM, is considerable, especially for *-ive* and *-ous*, amounting to roughly 13% in both cases. The picture is overall even worse for GPT-4. While the predictions for the high-regularity classes are good (almost always *-ity* for *-able* and *-ness* for *-ish*, like all other models), the match with humans is more than 10% worse than GPT-J for both *-ive* and *-ous*.

**Table 4.** Human evaluation

	MGL		GCM		LLMs	
Suffix	Type	Token	Type	Token	GPT-J	GPT-4
*-able*	1.000	1.000	1.000	1.000	0.893	0.960
*-ish*	1.000	1.000	1.000	1.000	0.997	1.000
*-ive*	0.720	0.680	0.760	0.700	0.632	0.440
*-ous*	0.560	0.520	0.640	0.520	0.503	0.400

The table shows the results of rule-based and exemplar-based analogy models as well as GPT-J and GPT-4 evaluated against human annotations. The measure is accuracy.

Why do GPT-J and GPT-4 match the human behavior so much worse than the much simpler type-based GCM? The key factor, we argue, is that both of these LLMs are driven by the token frequencies of the words in the training data. Just as for GPT-J, the token-based GCM and MGL models match the behavior of GPT-4 better than the type-based models (*SI Appendix*, Table S3). Token-oriented behavior is desirable in that it results in highly realistic implicit knowledge of individual words, as we have seen above. However, humans step back from the frequencies of individual words when making generalizations about possible words. LLMs seem to lack the ability to do this.

Furthermore, our results suggest that GPT-4’s overreliance on token frequency is if anything worse than that of GPT-J. Thus GPT-4’s morphological generalization behavior seems to be even less human-like than that of GPT-J. This finding is reminiscent of recently reported “inverse scaling effects,” more specifically the tendency of larger LLMs to rely even more strongly on prior statistics from the training data than smaller models do (86).

Since the performance of the best model, the type-based GCM, leaves room for improvement, we can ask why its performance was not better. The single biggest discrepancy was that the GCM selected *-ness* after *-ous* more than the humans did. Our analysis does not deal with the possibility that some people may consider the affix *-osity* to be a unified affix bundle, along the lines suggested in refs. 87 and 88; this would enhance its availability. Given that the GCM was fit to all word pairs attested in the Pile, the analysis also failed to allow for differences among human mental lexicons. Other studies have found considerable variability among human participants in the area of derivational morphology in general, and in preference for *-ity* over *-ness* specifically (89–93). In this context, it is noteworthy that the participants in our study were recruited from a highly educated community, whereas much of the Pile consists of web data such as informal discussions on Reddit (72). There is thus the possibility that there was a misalignment between the sociolect most strongly represented in the Pile and the ideolects sampled as part of our annotation study. Finally, the GCM works on the basis of word forms only, and has no way of taking into account similarities in meaning that also play a role in shaping morphological systems. In contrast, LLMs are able to consider similarities in meaning, but any advantage they might gain from their semantics in this highly focused task appears to be more than offset by the drawbacks of their reliance on token frequencies.

#### Familiarity of complex words.

Our results on nominalizations indicate that GPT-J and GPT-4 do not have a mental lexicon in the sense that humans do, in that they lack the ability to step back from word tokens and generalize over word types. Here, we present a brief demonstration that this observation pertains to morphologically complex words more generally, and not just to nominalizations. For this demonstration, we draw on the Hoosier Lexicon, a dataset of 19,320 English words that includes word frequencies and familiarity ratings on a seven-point Likert Scale (94). An important finding of the original study was a dissociation between word frequency and rated familiarity; one might expect the two to be highly correlated; however, some infrequent words are judged as much more familiar than their frequency would suggest. Needle et al. (95) identify morphological structure as an important factor contributing to this dissociation. A word like *precancellation*, with a recognizable prefix, stem, and suffix seems familiar even though it is rare, on the strength of the familiarity of its parts.

We analyze the *n* = 2,835 words in the Hoosier lexicon that have a frequency of less than 10,000 in the Pile (corresponding to a frequency of roughly 1 in 50,000,000 words or less). Leveraging the CELEX dictionary (54) and methodology from prior work (96, 97), we use affix-stripping to identify the n=1,005 words that exemplify derivational morphology by virtue of being parsable as a simpler word plus any combination of affixes. n=1,830 words cannot be parsed in this way, and we consider them to be simplex words (*SI Appendix*, Supporting Text). For human judgments, we take the familiarity ratings reported by Nusbaum et al. (94). We estimate the “familiarity” that GPT-J assigns to a word as the log probability that it assigns in the context of neutral prompts. Comparing log probabilities to human familiarity ratings is justified because the probabilities assigned to words by language models are known to correlate with psycholinguistic measures of lexical access (e.g., reading times; 98), which for humans are impacted by familiarity to a larger extent than frequency (99).

**Fig. 4.**
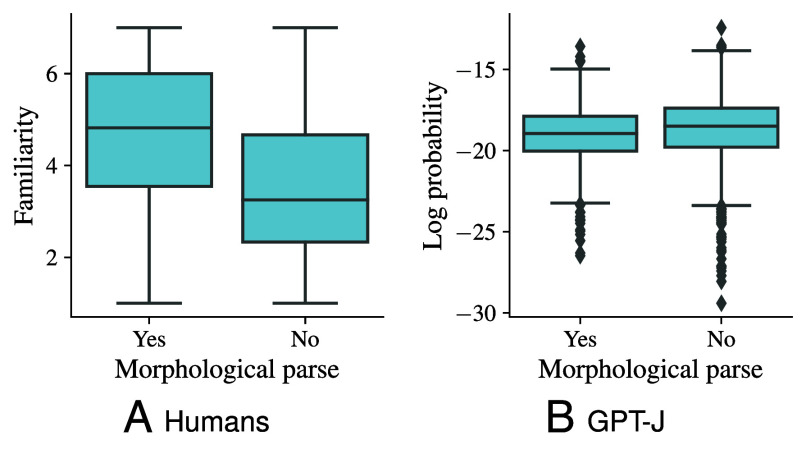
Impact of morphological decomposability of words on their familiarity as rated by human annotators (*A*) and the log probability assigned to them by GPT-J (*B*). Parsability increases familiarity for humans (*A*), but not for GPT-J (*B*).

Results for humans are displayed in Fig. 4*A*. The average familiarity of words with a morphological parse (n=1,005) is significantly higher than that of words with no morphological parse (n=1,830), t(2,120.2)=19.2, *P* < 0.001 (Welch’s *t* test). This confirms the results reported by Needle et al. (95). Due to this important factor, the correlation between familiarity and log frequency in the entire Hoosier lexicon proves to be modest according to a linear regression, F(1,19,318)=11,251.2, $R^{2}=0.368$, *P* < 0.001. For GPT-J, on the other hand, words with a morphological parse do not have any advantage (Fig. 4*B*); quite the opposite, the estimated familiarity of words with a morphological parse is significantly lower than the estimated familiarity of words with no morphological parse for GPT-J, t(2,285.9)=−4.9, *P* < 0.001. This outcome can be explained by the fact that the correlation between the log frequencies and the log probabilities assigned to the words by GPT-J is very high, F(1,19,318)=58,553.5, $R^{2}=0.752$, *P* < 0.001, and the target words without a parse have somewhat higher average frequency (m=4,285.1) than those having a parse (m=4,093.7).

Experimental studies on wordlikeness judgments (95) and on speech perception (100–102) show that humans continually monitor for known words inside rare or novel words. This means that their type-level lexical representations are exploited during processing, and can cause rare words to seem familiar. GPT-J does not rely on this type-level mechanism and hence lacks the dissociation between frequency and familiarity that is caused by morphological structure.

## Discussion

This paper provides empirical evidence for analogical linguistic generalization in LLMs. We found that an analogical cognitive model best explains how GPT-J nominalizes unseen nonce adjectives whose adjective class exhibits a high degree of variability. While the analogical and the rule-based model explained GPT-J’s predictions on adjective classes with a high degree of regularity equally well, we showed that the rule-like behavior for those adjectives is the end point of a continuum, where the position of an adjective class is precisely predictive from the level of heterogeneity in the training data. This result is in line with the predictions made by exemplar-based analogy models (e.g., ref. 25). It is not consistent with assuming rules, even with rules having a limited role, as in dual-mechanism approaches. We further found that GPT-J stores a considerable quantity of seen derivatives in its weights, again in line with analogical generalization.

Humans have been also argued to employ analogical generalization in adjective nominalization. However, while humans generalize based on types, we showed that GPT-J generalizes based on tokens. Is this difference between humans and LLMs reflected by their predictions? Indeed it is: The predictions of GPT-J, and similarly of GPT-4, are less human-like than any of the examined cognitive models. We further found that a central manifestation of type-level representations in humans, the decomposition of complex words into morpheme types, is not mirrored by language models. This suggests a critical difference in the organization of the lexicon between humans and LLMs. While humans have a mental lexicon organized around types, the lexical knowledge of LLMs is organized around tokens. Given that analogical generalization mechanisms depend on the lexicon they are operating on, a non-human-like lexicon leads to non-human-like generalizations.

We have presented an intensive study of a single morphological process, nominalization. However, our study has broader repercussions for the language sciences. In historical linguistics, a common trend is for the irregular forms in an inflectional paradigm to become regularized over time; typically, rare forms are affected first (83, 103, 104). However, in some cases, neologisms in a language take on the irregular form, and regular forms can even shift to become irregular (81, 105). In theories of language change, these phenomena are discussed under the rubric of analogical pressure; it is assumed that the acquisition, memory, or production of any inflected form is influenced by pressure from related forms in its neighborhood. Our exploration of frequency effects and neighborhood effects has shown that LLMs can and do operationalize analogical pressure. The boundary between morphology and syntax can be unclear, and in usage-based theories of linguistics, it is also proposed that multiword expressions can be stored in the mental lexicon (106). Krott (107) proposes an analogical approach to productive compounding, and our results suggest that an analogical theory implemented with a deep learning model could also hold promise for documented cases of variability in syntactic constructions (e.g., ref. 58).

Our findings are also related to the ongoing debate about how human-like the language skills of LLMs are (108–110), by highlighting a clear example of an area where the generalizations of LLMs—even the most performant ones—are decidedly non-human-like. The specific shortcoming that we observe in LLMs is that they do not distill token occurrences in text into more abstract type-level representations. From a semantics perspective, this can be interpreted as a failure to semantically ascend (111) to a level of representations that would make it possible for the LLMs to generalize over linguistic objects (specifically, word relations). More generally, this finding can be connected to converging evidence that LLMs fail to form meta-level representations the way humans do (112), in our case meta-level linguistic representations.

## Materials and Methods

### Cognitive Models.

The MGL (73, 74) works by inferring abstract rules from the lexicon. It starts by iterating over pairs of words and forming initial generalizations based on shared phonological features, which are then iteratively merged, yielding increasingly abstract rules. Each rule is associated with a value signifying its statistical reliability. The reliability is derived from the rate at which the rule applies to the *n* forms matching its structural description, adjusted for the uncertainty in the estimate of this rate due to the sample size *n*. To make predictions for a new input, the rule with the highest reliability that matches the phonological properties of the input is selected. In *SI Appendix*, Supporting Text, we provide example rules, including some induced by the MGL.

The GCM (29, 30, 75) does not infer abstract rules but instead stores all forms from the training data in an inventory. To make predictions for a new input, the input is compared to all instances that exhibit each relevant type of output (e.g., to all bases that have a derivative with *-ity*, vs. all bases that have a derivative with *-ness*). The selection of the output pattern is a cumulative effect of similarity and frequency (e.g., the number of different examples weighted by the similarity of those examples to the input). Because of the similarity-based weighting, a small number of highly similar examples can dominate a large number of less similar examples in the decision.

We use the implementation of MGL made available by Albright and Hayes (74), using default hyperparameters. For GCM, our implementation exactly follows prior studies in linguistics using the model (e.g., refs. 24, 27, and 74).

### Nonce Adjectives.

To create the nonce adjectives for the four adjective classes, we draw upon UniPseudo (76). UniPseudo uses an algorithm based on Markov chains of orthographic *n*-grams that it applies to a specifiable list of input words, generating a list of pseudowords. Importantly, when all input words end in a certain sequence of characters, the generated pseudowords also end in that sequence of characters. We leverage this property of UniPseudo to generate 50 nonce adjectives for each adjective class based on a curated list of adjectives drawn from CELEX (113), MorphoLex (114), and MorphoQuantics (115). For pseudoword length, we use the two most frequent lengths as measured on the extracted adjectives for each class and generate 25 pseudowords for each length. We use the bigram algorithm. See *SI Appendix*, Table S1 for the full list of pseudowords.

### GPT-J.

We describe the method we use to probe GPT-J more formally. Let *b* be a base (e.g., *sensitive*) and *s* be a suffix (e.g., *-ity*). We denote with d(b,s) the derivative resulting from adding *s* to *b* and applying all required morpho-orthographic changes (e.g., deletion of base-final e). For instance, for b=sensitive and s=−ity, we have d(b,s)=sensitivity. To measure the probability that GPT-J assigns to d(b,s) as a derivative of *b*, we use various prompts t(b). While some of the prompts ask GPT-J to nominalize *b* (e.g., t(b)=Turn thegiven adjectiveinto anoun.b→), others are less explicit (e.g., t(b)=b→). See *SI Appendix*, Supporting Text for the full set of prompts.

Given a filled prompt t(b), we pass it through GPT-J and measure the probability that GPT-J assigns to the two derivatives d(b,−ity) and d(b,−ness) as continuations of t(b).

We use the GPT-J implementation available on Hugging Face (116). GPT-J has a total of 6,053,381,344 parameters. All experiments are performed on a stack of eight GeForce GTX 1080 Ti GPUs (11 GB).

### Adjective Annotation.

For determining whether humans prefer *-ity* vs. *-ness* for the nonce adjectives, we collected human judgments from volunteers using the SoSciSurvey platform. Native speakers of English were recruited in a university community using snowball sampling. Hence most or all of them have university-level education. They were asked whether they were willing to participate in a survey about derivational morphology. They were unaware of the exact goals of the study. In total, 28 participants took part. Responses of six participants were removed because they did not finish the survey (attrition rate of 21.4%). Before starting the survey, the participants saw a consent form that described the study and explained that the anonymous responses would be collected, stored, and used for research purposes. They clicked “yes” to indicate their consent and continue on to the survey. The collection and use of the data were submitted to the institutional review board of the Allen Institute for AI for review. It ruled that the study was exempt from regulation because no personally identifiable information would be collected. See *SI Appendix*, Supporting Text for more details about the annotation study.

### GPT-4.

Since the OpenAI API does not provide access to output probabilities, we cannot use the same method as for GPT-J. Instead, we leverage GPT-4’s instruction-following capabilities and directly ask it which of the two derivatives it prefers for a given nonce adjective.

### Vocabulary Test.

To measure the probability that GPT-J assigns to a word *w*, we use various prompts t(w) (e.g., t(w)=Thefollowingisaword:w). See *SI Appendix*, Supporting Text for the full set of prompts.

Given a filled prompt t(w), we pass it through GPT-J and measure the probability that GPT-J assigns to the word.

## Supplementary Material

Appendix 01 (PDF)

## Data Availability

Outputs of computational models have been deposited in GitHub (117). Anonymized data on human judgments of nonwords collected in a crowd-sourcing experiment have been deposited in GitHub (117). The following data cannot be shared: One study reported in the article uses the Hoosier Lexicon dataset described in the study of Nusbaum et al. (94). This heavily cited work predates the FAIR standards by several decades. No online repository ever existed. Research groups using the data received it under a do-not-recirculate agreement. We feel that this situation does not compromise the replicability of our own work, because the study of Nusbaum et al. includes ample detail about how the data were collected, and its main claims have been independently validated by other laboratories. Previously published data were used for this work: CELEX: (113). MorphoLex: (114). MorphoQuantics: (115).
