# Supplementary material for: Derivational morphology reveals analogical generalization in large language models
Source: Proc Natl Acad Sci U S A. 2025 May 9;122(19):e2423232122. doi: 10.1073/pnas.2423232122 (PMC12088417; doi:10.1073/pnas.2423232122)
Supplement: Supplementary file 1 — Appendix 01 (PDF) [file pnas.2423232122.sapp.pdf]

Supporting Information for

Derivational Morphology Reveals Analogical Generalization in Large Language Models

Valentin Hofmann, Leonie Weissweiler, David R. Mortensen, Hinrich Schütze, Janet B. Pierrehumbert

To whom correspondence may be addressed. Email: valentinh@allenai.org or janet.pierrehumbert@oerc.ox.ac.uk.

This PDF file includes:

Supporting text
Figs. S1 to S3
Tables S1 to S3
SI References

Supporting Information Text

Example Rules. The standard notation for linguistic rules is as follows:

$$\text{SD} \rightarrow \text{SC} \ / \ \text{LC} \ \_\_ \ \text{RC}$$

Here, SD is the structural description of the rule, SC specifies the change to produce the output, LC is an optional left-hand context, and RC is an optional right-hand context.

Many researchers might suggest the following default rule for the phonological spell-out of nominalization. NOM represents the underlying morpheme that may be spelled out as *-ness* or *-ity* at the phonological level. (Throughout the paper we make the simplifying assumption that the two spell-outs are synonymous.)

$$\text{NOM} \rightarrow \textit{-ness}$$

This rule has no left or right context because the morpheme NOM would only occur at the morphosyntactic level on stems with suitable syntactic and semantic properties. Under this assumption, all forms in *-ity* would be memorized exceptions. The most statistically reliable rule for our dataset — and one that is induced by the MGL — includes a specification of the left context:

$$\text{NOM} \rightarrow \textit{-ness} \ / \begin{Bmatrix} \textit{-ed} \\ \textit{-ing} \\ \textit{-ish} \\ \textit{-less} \end{Bmatrix} \_\_$$

This rule corresponds to the blue dots in Fig. 3 in the main article. The following rule for R-ITY is also induced by the MGL but is less reliable:

$$\text{NOM} \rightarrow \textit{-ity} \ / \begin{Bmatrix} \textit{-able} \\ \textit{-al} \\ \textit{-ar} \\ \textit{-ic} \end{Bmatrix} \_\_$$

This rule corresponds to the orange dots in Fig. 3 in the main article.

Prompts. We want to test which of two derivatives — the one ending in *-ness* or the one ending in *-ity* — is preferred by a language model. To do so, we need to measure the probability that the language model assigns to the two competing forms. For example, we need to measure the probability that the language model assigns to *sensitiveness*, and the probability that it assigns to *sensitivity*. Language models such as GPT-J and GPT-4 always assign probabilities to tokens *given a sequence of preceding tokens*. Therefore, in order to measure the probability that a language model assigns to a specific derivative, we need to decide on what tokens to use as the preceding context. This is commonly referred to as *prompting*, and the sequence of preceding tokens that is fed into the language model as *prompt* (1). Properties of the prompt (e.g., the exact wording of a request) can substantially affect the language model predictions (2), which is why it has become common practice to examine several different prompts when analyzing the behavior of language models. Here, we use the following 12 prompts to measure the probabilities that GPT-J assigns to the derivatives:

- *Nominalized adjective:*
- *Noun:*
- *The following is a nominalized adjective:*
- *The following is a noun:*
- $b \rightarrow$
- *b :*
- *b -*
- *b*
- *Adjective: b Nominalization:*
- *Form the nominalization of the given adjective.* $b \rightarrow$
- *Nominalize the given adjective.* $b \rightarrow$
- *Turn the given adjective into a noun.* $b \rightarrow$

As in the main text, *b* here is a variable that refers to a base. For example, with the prompt *Nominalized adjective:* and the base *sensitive*, we measure the probability assigned to *sensitivity* in the context *Nominalized adjective: sensitivity* as well as the probability assigned to *sensitiveness* in the context *Nominalized adjective: sensitiveness*. The presented results are averaged across prompts; for example, to get GPT-J's match with the cognitive models, we calculate the match based on each of the 12 prompts and report the mean of these 12 scores.

We use the following prompts to measure the probabilities that GPT-J assigns to the words in the vocabulary test:

- *Word:*
- *Real word:*
- *The following is a word:*
- *The following is a real word:*

Derivative Statistics. We analyze the statistics of *-ity* and *-ness* derivatives in the Pile (Table S2). We first focus on *type frequency*, i.e., the number of different derivatives contained in the Pile. For most classes, there is a clear preference for either *-ity* or *-ness*, the only two exceptions being adjectives ending in *-ive* and *-ous*. For adjectives ending in the Germanic suffixes *-ed*, *-ing*, *-ish*, and *-less*, there is a particularly strong preference for *-ness*, although a few derivatives in *-ity* can be found in the data. These statistics are similar to the results of a recent analysis based on dictionary data (3), indicating that the Pile provides a realistic picture of the variation between *-ity* and *-ness* in present-day English.

Next, we turn to *token frequency*, i.e., the number of times individual derivatives occur in the Pile. We notice that the trends for type frequency are largely reflected by token frequency: in the case of adjective classes for which *-ity* derivatives have a higher type frequency than *-ness* derivatives, *-ity* derivatives also tend to have a higher average token frequency than *-ness* derivatives (and vice versa). The only exception is *-ous*, where *-ity* has a lower type frequency but a higher average token frequency than *-ness*. This is due to a particularly large number of *-ity* derivatives in the high token frequency range: excluding the top 5% of derivatives with the highest token frequency, the average token frequency is higher for *-ness* (73.0) than *-ity* (15.1), in line with the type frequency trend for *-ous*.

Finally, we examine a measure that linguistic scholarship has suggested to be particularly relevant for productivity (4, 5), specifically the number of *hapaxes* (i.e., derivatives occurring only once in the Pile). Here, the trends for individual adjective classes are similar to type frequency and token frequency, with the potential exception of *-ive*, where the preponderance of *-ity* compared to *-ness* is slightly less pronounced.

Adjective Annotation. Each participant coded half of the nonce words (i.e., 100 nonce words). The 22 participants who completed the full survey were evenly divided between the two halves. Participants were first shown an introductory message explaining the task as shown in Fig. S1a. Participants who consented to the collection and use of their data, as described in the introductory message, indicated their consent by clicking "yes". They were then given one of two survey versions, each with 100 nonce words that cycled through the four suffixes to avoid repetition. To reduce the total time necessary for completing the survey, participants were immediately shown the next question upon clicking a word. An example of a question is shown in Fig. S1b.

Fig. S2b plots for each tested adjective class the ratio of bases for which participants overall preferred *-ness* over *-ity*, i.e., more participants selected the *-ness* rather than the *-ity* derivative. There is a clear preference for *-ity* in the case of *-able* and a clear preference for *-ness* in the case of *-ish*. For the two suffixes with a larger degree of competition, *-ive* shows the expected pattern, with participants preferring *-ity* over *-ness* for the majority of bases, but *-ous* shows a preference for *-ity*, which is different from its greater association with *-ness* in the Pile. This can also be seen from the ratio of participants preferring *-ness* over *-ity* for individual bases (see Fig. S3a), which is on average smaller than 50% for *-able* (17.7%), *-ive* (39.8%), and *-ous* (47.5%), and greater than 50% only for *-ish* (95.1%). Fig. S3a also shows a high degree of variation between individual bases of a certain adjective class: e.g., for *-ous*, there are bases for which participants clearly preferred *-ity* (e.g., 81.8% preferred *-ity* for *indaminous*), but there is also a base for which participants exclusively selected *-ness* (100% preferred *-ness* for *rebelorous*).

Participants differed in terms of how often they selected *-ity* or *-ness* for each adjective class (see Fig. S3b). For example, 13 participants preferred *-ity* for *-ous* bases, but nine participants preferred *-ness*. This high degree of variation is reflected by a small inter-annotator agreement (IAA) of 0.335, measured using Fleiss' κ. However, measuring IAA on all bases hides the fact that IAA is substantially higher for *-ish* (0.899) and *-able* (0.587) than for *-ive* (0.096) and *-ous* (0.054), measured using Gwet's AC1 (6). There is also a correlation between the responses given by individual participants for bases of different adjective classes, especially between *-able* and *-ive* (0.417), and *-ive* and *-ous* (0.415), measured using Pearson's r.

Morphological Parse. The parsability of words in the Hoosier lexicon is determined as follows. In a first step, we check whether a word is contained in CELEX (7), a lexical database that contains information about the morphological status of more than 50,000 English words. 16,417 words from the Hoosier lexicon are listed in CELEX. For the remaining 2,903 words, we determine the morphological status by means of a simple method from prior work (8, 9): we test whether the beginning or end of words matches common prefixes/suffixes of the English language, and whether the remaining part of the word is a stem. To do so, we draw upon a list of 46 English prefixes and 44 English suffixes (10). As potential stems, we use all English words contained in CELEX. The algorithm is sensitive to morpho-orthographic rules of English (11).

As a result of this procedure, 6,499 words from the Hoosier lexicon are classified as morphologically complex. The words are diverse in terms of the involved affixes: except for *pseudo* and *mini*, all affixes from the list mentioned above show up.

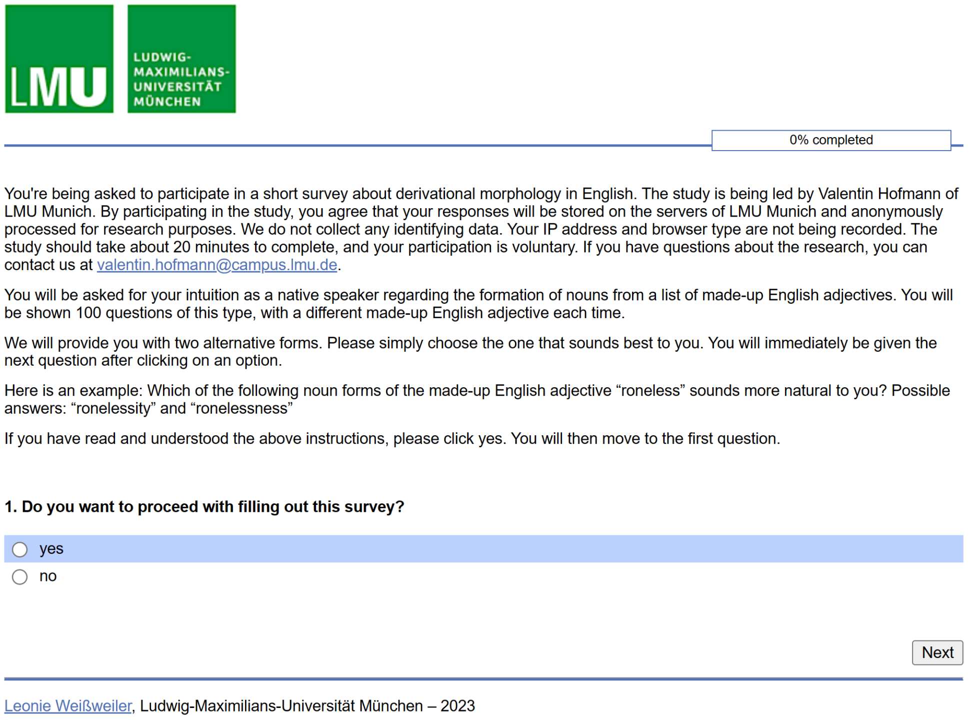

(a) Introductory message

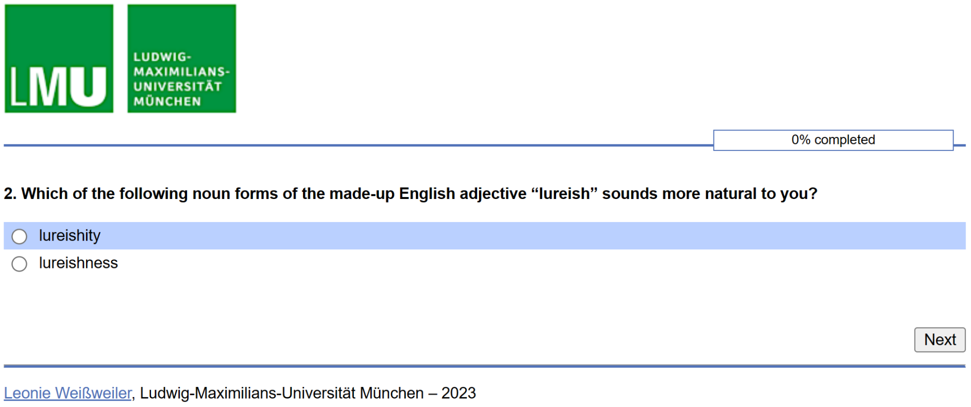

(b) Example question

Fig. S1. Screenshots of the introductory message seen by participants of our survey (a) and an example question given to participants (b).

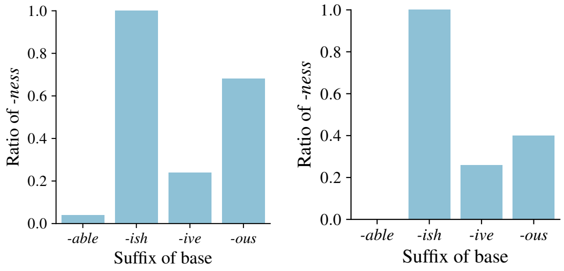

(a) GPT-J **(b)** Humans

Fig. S2. Distribution of preferred nominalization type (specifically, ratio of *-ness* derivatives) for unseen nonce adjectives, for GPT-J (a) and human annotators (b). The ratio is computed as the number of *-ness* predictions divided by the total number of predictions. Panel (a) replicates Fig. 1e from the main article for easier comparison.

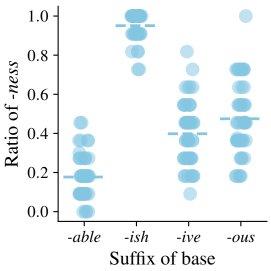

(a) Base variation

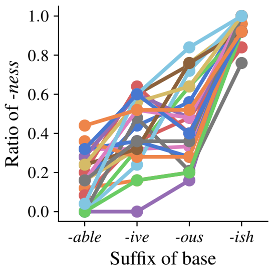

(b) Participant variation

Fig. S3. Variation in the derivative preferred by humans, shown separately for bases (a) and participants (b). In (a), each dot represents one base. In (b), each line represents the response pattern of one participant in our annotation study.

Table S1. Complete list of all used nonce adjectives.

-able	*-ish*	*-ive*	*-ous*
actignable	*badyish*	*atecusive*	*adodagious*
anilicable	*beavish*	*cogective*	*adupendous*
anvastable	*breyish*	*conovative*	*anoninous*
chalinable	*carmish*	*cormasive*	*aurtiguous*
comfolvable	*clangish*	*cuminitive*	*cazardous*
compechable	*clurlish*	*decertive*	*coivonous*
condumable	*cunkish*	*deflosive*	*creninous*
contaitable	*devevish*	*defrertive*	*dardulous*
corgervable	*direish*	*dejovative*	*dexarious*
covornable	*doutish*	*depulsive*	*erenymous*
cresucable	*dwaplish*	*dermasive*	*eretulous*
enocutable	*fadyish*	*dignitive*	*euphitious*
expeaceable	*fawkish*	*dimusitive*	*eutrigeous*
expelocable	*fevetish*	*exhauctive*	*faluminous*
expernable	*fevewish*	*expecative*	*fapturous*
fispoceable	*fevilish*	*extuctive*	*glamalous*
fupeactable	*frietish*	*gederative*	*glumonous*
fusuperable	*friquish*	*imimative*	*gluninous*
imalatable	*ghumpish*	*impuctive*	*gropenious*
impalvable	*gireish*	*indetative*	*hibeguous*
inbeadable	*goguish*	*nogensive*	*honoderous*
inedifiable	*higetish*	*nombasive*	*indaminous*
infoustable	*knarish*	*nonvuptive*	*iniragious*
intoundable	*laretish*	*nutensive*	*insicious*
intountable	*lureish*	*obsensive*	*lasavenous*
inveicable	*lurmish*	*peditative*	*leamogous*
irediocable	*moguish*	*pedulsive*	*ligegious*
mecoushable	*peftish*	*pepulative*	*liratonous*
parendable	*preanish*	*pransitive*	*luticorous*
peplaicable	*prienish*	*prediasive*	*malicinous*
praleckable	*purerish*	*prititive*	*meglarious*
preneckable	*radyish*	*protrative*	*momogorous*
prequakable	*reckish*	*pumbative*	*mystuorous*
previnable	*redyish*	*recentive*	*nomeneous*
previtable	*rourfish*	*recumotive*	*oblicious*
puneadable	*shigeish*	*rejeptive*	*pecacious*
pustameable	*skierish*	*ruchontive*	*plalorous*
redeptable	*slarish*	*seceptive*	*poncorous*
rempadable	*slownish*	*sejensive*	*prolacious*
retaleable	*slundish*	*serposive*	*ralygerous*
sempoivable	*slungish*	*submiative*	*ravarious*
swimitable	*snoulish*	*submictive*	*reamorous*
tegornable	*sonkish*	*submistive*	*rebelorous*
unaclerable	*tivilish*	*sumpertive*	*slaicitous*
unalintable	*turgeish*	*sumurative*	*suspibious*
undeperable	*wabyish*	*suprective*	*tefigious*
unutintable	*waguish*	*tecensive*	*trospurous*
unvatrable	*wainish*	*tendusive*	*undicitous*
unvediable	*wawkish*	*tredictive*	*vexuteous*
utililable	*woungish*	*vederative*	*vombageous*

Table S2. Statistics of *-ity* and *-ness* derivatives for the 10 examined adjective classes in the Pile (12), the corpus used to train GPT-J (13). The total number of bases is 48,995. The values for token frequency are averaged across all word types belonging to a specific adjective class.

	Type frequency		Token frequency		Hapaxes	
Suffix	*-ity*	*-ness*	*-ity*	*-ness*	*-ity*	*-ness*
-able	11,081	1,034	3937.7	817.3	1,673	226
-al	9,133	1,011	5904.9	172.1	2,078	251
-ar	2,433	214	5833.7	10.3	451	59
-ed	62	4,786	2.4	539.6	28	1,134
-ic	6,215	617	4162.7	45.7	790	175
-ing	2	1,600	1.0	1104.5	2	448
-ish	0	1,502	0.0	397.0	0	437
-ive	4,508	2,438	15075.8	3252.1	626	554
-less	3	2,020	1.7	1159.8	1	506
-ous	1,372	2,450	5453.1	2420.3	325	675

Table S3. Match of rule-based and exemplar-based models with GPT-4 on nonce adjectives.

	MGL		GCM	
Suffix	Type	Token	Type	Token
-able	.960	.960	.960	.960
-ish	1.000	1.000	1.000	1.000
-ive	.400	.480	.440	.500
-ous	.680	.760	.640	.800

References

1. P Liu, et al., Pre-Train, Prompt, and Predict: A Systematic Survey of Prompting Methods in Natural Language Processing. *ACM Comput. Surv.* **55**, 1–35 (2023).
2. JW Rae, et al., Scaling Language Models: Methods, Analysis & Insights from Training Gopher. Preprint, arXiv 2112.11446 (2022).
3. S Arndt-Lappe, Analogy in Suffix Rivalry: The Case of English -Ity and -Ness. *Engl. Lang. & Linguist.* **18**, 497–548 (2014).
4. H Baayen, R Lieber, Productivity and English Derivation: A Corpus-Based Study. *Linguistics* **29**, 801–844 (1991).
5. RH Baayen, A Renouf, Chronicling the Times: Productive Lexical Innovations in an English Newspaper. *Language* **72**, 69–96 (1996).
6. KL Gwet, Computing Inter-Rater Reliability and Its Variance in the Presence of High Agreement. *Br. J. Math. Stat. Psychol.* **61**, 29–48 (2008).
7. RH Baayen, R Piepenbrock, L Gulikers, *The CELEX Lexical Database (CD-ROM).* (Linguistic Data Consortium, Philadelphia, PA), (1995).
8. V Hofmann, J Pierrehumbert, H Schütze, "Predicting the Growth of Morphological Families from Social and Linguistic Factors" in *Proceedings of the 58th Annual Meeting of the Association for Computational Linguistics.* pp. 7273–7283 (2020).
9. V Hofmann, H Schütze, J Pierrehumbert, "A Graph Auto-Encoder Model of Derivational Morphology" in *Proceedings of the 58th Annual Meeting of the Association for Computational Linguistics.* pp. 1127–1138 (2020).
10. D Crystal, *The Cambridge Encyclopedia of the English Language.* (Cambridge University Press, Cambridge, UK), (1997).
11. I Plag, *Word-Formation in English.* (Cambridge University Press, Cambridge, UK), (2003).
12. L Gao, et al., The Pile: An 800GB Dataset of Diverse Text for Language Modeling. Preprint, arXiv 2101.00027 (2020).
13. B Wang, A Komatsuzaki, GPT-J-6B: A 6 Billion Parameter Autoregressive Language Model. https://github.com/kingoflolz/mesh-transformer-jax (2021).
